# The Multi‐Kinase Inhibitor GZD824 (Olverembatinib) Shows Pre‐Clinical Efficacy in Endometrial Cancer

**DOI:** 10.1002/cam4.70531

**Published:** 2024-12-30

**Authors:** Dongli Liu, Dylan Glubb, Tracy O'Mara, Caroline E. Ford

**Affiliations:** ^1^ Gynaecological Cancer Research Group, Lowy Cancer Research Centre, School of Clinical Medicine, Faculty of Medicine & Health University of New South Wales Sydney New South Wales Australia; ^2^ Cancer Research Program QIMR Berghofer Medical Research Institute Herston Queensland Australia

## Abstract

**Objective:**

Endometrial cancer is one of the few cancers for which mortality is still increasing. A lack of treatment options remains a major challenge, particularly for some subtypes of the disease. GZD824, also known as *olverembatinib*, is a multi‐kinase inhibitor previously investigated in clinical trials for chronic myeloid leukaemia and Ph+ acute lymphoblastic leukaemia as a BCR‐ABL inhibitor. This study aimed to investigate the pre‐clinical efficacy of GZD824 for the treatment of EC.

**Methods:**

Here, we undertook pre‐clinical evaluation of GZD824 in seven endometrial cancer cell lines (HEC‐1‐A, HEC‐1‐B, MFE296, RL95‐2, Ishikawa, KLE and ARK‐1), one normal immortalised endometrium derived cell line (E6E7hTERT) and primary mesothelial and fibroblast cells isolated from normal omentum samples.

**Results:**

GZD824 inhibited the proliferation of all endometrial cancer cell lines, which were significantly more sensitive to GZD824 compared to normal cells (*p* = 0.030). GZD824 significantly inhibited migration in Ishikawa (endometrioid) and ARK1 (serous) endometrial cancer cell lines and significantly inhibited invasion in the ARK1 cells. Whole transcriptome regulation following two doses (0.1 and 1 μM) of GZD824 in Ishikawa and ARK1 cells was investigated via RNA‐seq, and key components of enriched pathways were investigated at the translational level. Key pathways altered included ROR1/Wnt, GCN2‐ATF4, epithelial to mesenchymal transition (EMT) and PI3K‐AKT.

**Conclusion:**

Together, these studies support further investigation of GZD824 as a potential therapeutic agent in endometrial cancer, potentially in combination with immune checkpoint inhibitors.

## 1 Introduction

Endometrial cancer (EC) is the sixth most prevalent malignancy worldwide and is one of the few cancers with increasing mortality [1]. Although EC patients generally have a good outcome and are often cured by hysterectomy and bilateral salpingo‐oophorectomy ± adjuvant therapy, some patients with advanced‐stage or high‐risk histologies receive upfront chemotherapy. Furthermore, relapse rates reach 20% in cases with the endometrioid histological subtype and 50% in non‐endometrioid (e.g., serous and clear cell) subtype cases [2]. Indeed, women who are diagnosed with non‐endometrioid tumour subtypes or recurrent disease have a poor prognosis [2].

Current treatment options for patients with advanced disease are limited, with only five drugs approved for EC therapy by the Food and Drug Administration (USA). The current first‐line standard chemotherapy is the carboplatin‐paclitaxel doublet. However, the PORTEC‐3 clinical trial showed only patients harbouring *TP53* mutations (mostly tumours of the serous subtype) benefited from adjuvant chemoradiotherapy with this doublet, compared to radiotherapy alone [3]. There has been progress for other prognostic molecular subtypes recently, including MMR‐deficient (MMRd) or microsatellite instability high (MSI‐H) tumours for which the immune checkpoint inhibitor dostarlimab‐gxly in combination with carboplatin and paclitaxel has been approved for primary advanced or recurrent patients [4, 5]. Furthermore, the multi‐kinase inhibitor lenvatinib combined with pembrolizumab has shown efficacy in a recent clinical trial regardless of MSI or MMR status [6] and been approved in non‐MMRd/MSI‐H recurrent EC patients [7]. In addition, no treatment has been approved specifically for the patients characterised with the p53 wildtype molecular subtype, leaving a gap in addressing the needs of the 70% of MMR‐proficient patients falling into this category.

We previously identified the receptor tyrosine kinase‐like orphan receptor—ROR1, as a prognostic biomarker via an Australian population‐based EC patient cohort (*n* = 499) [8]. Additionally, we observed that silencing *ROR1* resulted in the inhibition of cell proliferation in the KLE EC cell line [8, 9]. These observations suggest that ROR1 is a potential therapeutic target in EC. A recent study analysed the structure and dynamics of Wnt binding receptor tyrosine kinases and proposed two multi‐kinase inhibitors, ponatinib and GZD824 (also known as HQP1351 or olverembatinib) [10]. Most previous ROR1 therapies have targeted extracellular regions of ROR1 [11, 12], but these small molecules are thought to impair scaffolding functions of ROR1 upon binding to the tyrosine kinase domain, consequently inhibiting ROR1 signalling [10, 13]. Ponatinib was approved as a second‐line treatment for patients with Ph+ acute lymphoblastic leukaemia (ALL) and chronic myeloid leukaemia (CML) as a BCR‐ABL inhibitor, with a particular focus on addressing the ABL‐T1351 mutation [14, 15]. In the context of EC, ponatinib has been shown to inhibit the growth of FGFR2 mutant tumours [16, 17]. However, a post‐marketing amendment was issued to optimise the dosing of ponatinib in chronic‐phase CML due to adverse events associated with the drug [18]. In comparison, GZD824, which was also identified as a BCR‐ABL inhibitor originally, has shown to be well‐tolerated while effectively inhibiting tumour growth in CML patients in Phase I/II clinical trials [19, 20, 21].

GZD824 has also been found to inhibit the GCN2‐ATF4 pathway and sensitise cancer cells to amino acid starvation stress [22]. Notably, GCN2 is encoded by *EIF2AK4*, a potential EC susceptibility gene identified through follow‐up of genome‐wide association studies (GWAS) [23, 24]. Further highlighting its therapeutic potential in EC treatment, greater expression of *EIF2AK4 was* predicted by a transcriptome‐wide association study to increase EC risk [25]. Given the ability of GZD824 to target ROR1 and GCN2, we investigated the effect of GZD824 on EC cell lines and explored the underlying mechanisms through gene expression analyses.

## 2 Methodologies

### 2.1 Cell Culture

EC cell lines HEC‐1‐A, HEC‐1‐B, RL95‐2, KLE, MFE296, Ishikawa and ARK‐1 as well as the normal immortalised endometrial cell line E6E7hTERT and healthy omentum‐derived human peritoneal mesothelial (HPMC) and Normal Omentum Fibroblast (NOF) [26] were included in this study. HPMCs and NOFs were derived from patients treated at the Royal Hospital for Women, Sydney, with informed consent and under ethics approval #16/108 by South Eastern Sydney Local Health District Human Research Ethics Committee (HREC). HEC‐1‐A, HEC‐1‐B and RL95‐2 were gifts from Professor Deborah Marsh (UTS, Australia), MFE296 was a gift from Associate Professor Kyle Hoehn (UNSW, Australia), Ishikawa was kindly provided by Professor Jeff Holst (UNSW, Australia) and ARK‐1 was provided by Dr. Alessandro Santin (Yale University, USA). Cell lines were maintained in medium containing 10% foetal bovine serum, 1% GlutaMAX and 1% penicillin/streptomycin and cultured in 5% CO_2_ at 37°C. Specifically, Ishikawa and MFE296 were cultured in MEM media, ARK‐1 was cultured in RPMI media, HEC‐1‐A was grown in McCoys 5A media supplemented with 2.2 mM sodium pyruvate, HEC‐1‐B was grown in MEM media with extra non‐essential amino acids and 1 mM sodium pyruvate, RL95‐2 was cultured in DMEM/F12 with additional 0.005 mg/mL insulin, HPMC and NOF were cultured in antibiotic free DMEM with additional non‐essential amino acids and E6E7hTERT was cultured in DMEM media. EC cell lines underwent regular mycoplasma testing and were validated at the cell line identification service at the Garvan Institute of Medical Research (Sydney, Australia).

### 2.2 Drug Sensitivity Assay

Cell lines were seeded in 12‐well tissue culture plates for 24 h, followed by treatment with GZD824 (serial dilutions spanning from 0 to 100 μM). Cell coverage was monitored using the IncuCyte S3 Live Cell Analysis System for a duration of 72 h. Phase contrast images were captured at 3 h intervals using a 10× objective lens. The cell confluence at each timepoint was normalised against that of the baseline. Cell viability at 72 h post treatment was assessed via the cell counting kit‐8 (CCK‐8, Sigma‐Aldrich, USA) as per the manufacturer's protocol.

### 2.3 Wound Healing Assay

Ishikawa and ARK1 were selected for the functional analysis of GZD824 treatment. Ishikawa serves as a well‐established model for Type I EC, characterised by well differentiated endometrial adenocarcinoma pathology and positive estrogens and progesterone receptor expression. ARK1 is one of the few robust models for high grade serous EC (Type II EC). Wound healing assay was performed using the IBIDI culture insert system (IBIDI, USA) as per manufacturer's protocol. EC cells were plated for 24 h before the culture inserts were removed. The cells were then treated with GZD824 at 0.1 μM or vehicle control, and photographs of the plates were taken at the base line (time 0), 24 and 48 h and analysed using TScratch software (ETH Zurich, Switzerland) [27]. Three independent experiments were conducted, each incorporating two technical replicates.

### 2.4 Transwell Migration and Invasion Assay

EC cells were treated with GZD824 at 0.1 μM or vehicle control for 24 h and then subjected to migration or invasion assay via Corning transwell inserts or Matrigel pre‐coated transwell inserts (Corning Life Sciences, USA), as previously described [28]. Ishikawa cells were incubated for 48 h for both assays, while ARK1 cells were plated for migration or invasion for 24 h following transfection. Three independent experiments were conducted, each incorporating two technical replicates.

### 2.5 RNA‐Seq

Two independent clones of Ishikawa or ARK1 cells were incubated with 0.1 μM, 1 μM GZD824 or vehicle control for 48 h. Total RNA was extracted from cells with RNeasy Mini kit (Qiagen, USA) and treated with DNase I (Thermofisher, USA). Libraries were prepared using the Illumina Stranded mRNA Prep Ligation kit. Samples were sequenced with the NextSeq 500 platform (1 × 75bp) at the Ramaciotti Centre for Genomics, UNSW. QC reports were generated with FastQC (v 0.11.8) [29] and viewed with MultiQC (v 1.7) [30]. Sequenced reads were then aligned using STAR (v 2.7.2b) [31] against the reference human genome (GRCh38.p13) and counted at the gene level using featureCounts [32]. Differential expression analysis was performed using edgeR (v 3.28.1) [33]. |Log fold change| > 0.6 and false discovery rate (FDR) < 0.05 was set as cut‐off for differentially expressed genes. KEGG pathway enrichment analysis was performed using Clusterprofile (v 3.18.1) [34]. Gene Set Enrichment Analysis (GSEA) was performed using the GSEA software (v 4.3.3) [35]. The hallmark gene sets were selected. Significance was set at FDR *q* < 0.25. The online gene annotation tool Metascape (v3.5) [36] was applied to build the protein–protein interactions (PPIs) network. The Molecular Complex Detection (MCODE) algorithm was applied to identify key clusters of genes for those PPI networks that contain more than three nodes. In addition, differentially expressed genes from the different conditions were analysed using Ingenuity Pathway Analysis software (QIAGEN Inc., https://www.qiagenbioinformatics.com/products/ingenuitypathway‐analysis). Pathway and disease/function enrichment analyses were conducted, and significance values (*p*‐values of overlap) were determined using the right‐tailed Fisher's Exact Test. The overall activation/inhibition states of canonical pathways were predicted based on a *z*‐score algorithm. Upstream regulator analysis was performed to identify molecules that potentially explain the observed patterns of differential gene expression and statistical significance determined using the right‐tailed Fisher's Exact Test. The changes in the expression of upstream regulator targets were compared to expected causal effects from the Ingenuity Knowledge base, and a *z*‐score was calculated to assess the activation state of the upstream regulator.

### 2.6 Western Blot

Total protein lysates of the cells were made with cell lysis buffer (Cell Signalling Technology, USA) containing protease/phosphatase inhibitor cocktail (Cell Signalling Technology, USA). Total protein lysate was loaded for western blot analysis, as previously described [37]. The antibodies used were ROR1 (Cell Signalling Technology, #4102), E‐cadherin (Cell Signalling Technology, #3195), Vimentin (Cell Signalling Technology, #5741), ATF4 (Cell Signalling Technology, #11815), cleaved PARP (Cell Signalling Technology, # 5625), pAKT (Cell signalling Technology, #4060), AKT (Cell Signalling Technology, #4691) and α‐Tubulin (Cell Signalling Technology, #3873). All markers were run three times to verify trends in protein expression.

### 2.7 Statistical Analysis

The impact of GZD824 on cell confluence was assessed using a two‐way ANOVA test (doses and time as factors), corrected for multiple testing using the Bonferroni method. The half inhibitory concentration (IC50) for each cell line was obtained from dose–response curves, using a non‐linear dose–response curve fitting analysis (log inhibition vs. normalised response‐variable slope). Differences of drug sensitivity between normal cells and EC cell lines and between GZD824‐treated and vehicle control samples in in vitro assays were analysed using the Mann–Whitney *U* test (two‐tailed). All the statistical analyses were performed in GraphPad Prism (v9.0.0) with significance set as *p* < 0.050. Data was presented as mean ± standard deviation.

## 3 Results

### 3.1 GZD824 Inhibited EC Cell Proliferation in a Dose‐Dependent Manner

**FIGURE 1:**
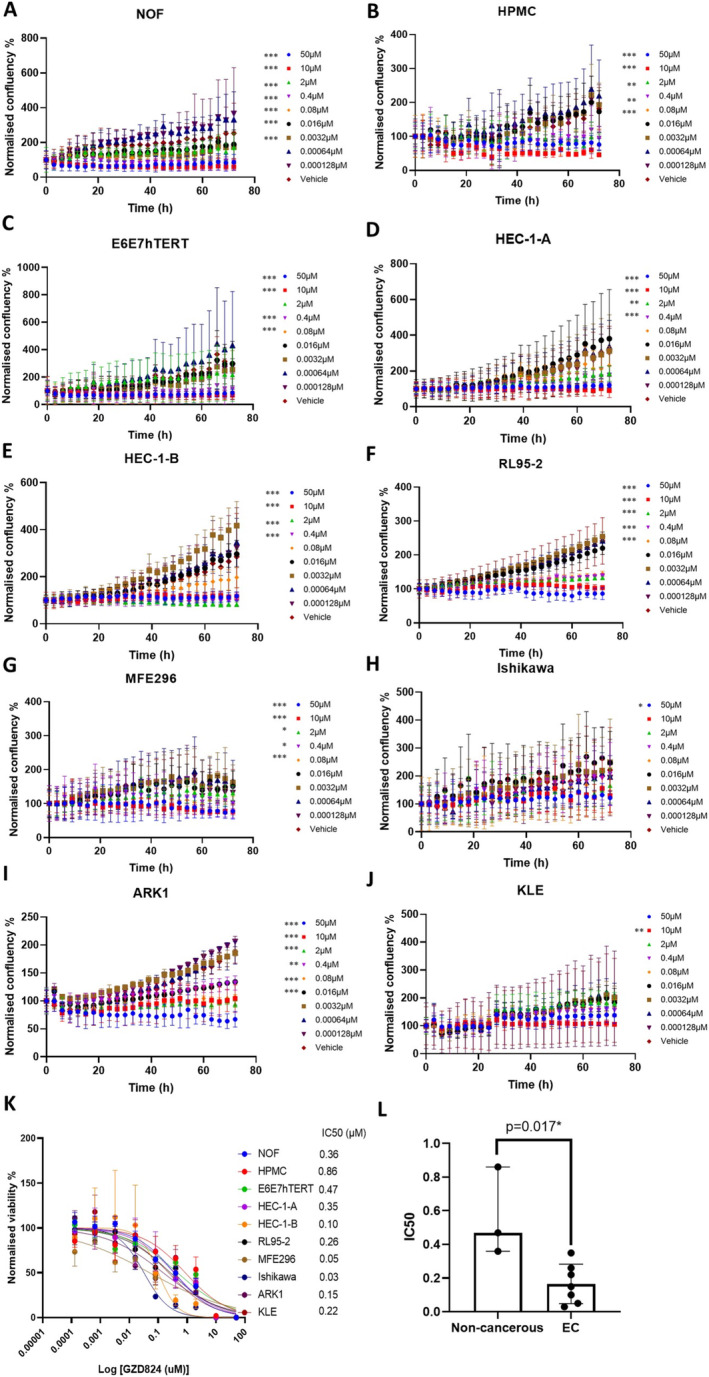
Endometrial cancer cells are more sensitive to GZD824 compared to non‐cancerous cells. (A–J) Confluence of cell lines following a series of GZD824 treatments over a total of 72 h incubation in the IncuCyte S3 system. Two‐way ANOVA with Bonferroni correction for multiple comparisons using test was applied to compare each dose to vehicle control. *adjusted *p* < 0.05, **adjusted *p* < 0.01, ***adjusted *p* < 0.001. (K) Dose response curves of GZD824 in normal immortalised EC cell line E6E7hTERT, omentum‐derived cells HPMC and NOF and endometrial cancer cell lines at 72 h. (L) Compared to the non‐canerous cells (HPMC, NOF and E6E7hTERT), endometrial cancer cell lines showed a significant lower mean IC50 value in response to GZD824 treatment (*p* = 0.017). *p < 0.05. For all panels *n* = 3, error bars represent standard deviation of the mean.

We performed dose response analysis in seven EC cell lines (Table 1), an immortalised normal endometrial cell line (E6E7hTERT) and omentum‐derived HPMC and NOF cells. The E6E7hTERT and the two primary cell types (HPMC and NOF) found in the omentum were included as models of non‐cancerous peritoneal cell lines. To assess the impact of GZD824 on the growth of the cell lines, we employed the IncuCyte S3 Live Imaging platform for monitoring real‐time cell confluence over a 72 h duration following a series of GZD824 treatments. Notably, a significant decrease in cell confluence was observed for most of the cell lines with doses over 80 nM (Figure 1A–J). Next, we assessed the cell viability at the end time point (72 h) to estimate the IC50 of the 10 cell lines by fitting non‐linear dose–response curves (Figure 1K). Compared to the non‐tumoural cells, EC cell lines were more sensitive to GZD824 with a significantly lower mean IC50 value (166 ± 117 vs. 563 ± 263 nM, *p* = 0.017, Figure 1L).

**TABLE 1:** Summary of endometrial cancer cell lines.

Cell line	Histopathological type
HEC‐1‐A [38]	Grade 2 endometrial adenocarcinoma
RL95‐2 [39]	Grade 2 moderately differentiated adenosquamous carcinoma of the endometrium
Ishikawa [40]	Grade 1 endometrial adenocarcinoma
ARK1 [41]	Uterine papillary serous adenocarcinoma
MFE296 [42]	Moderately differentiated endometrial adenocarcinoma
KLE [43]	Poorly differentiated endometrial adenocarcinoma
HEC‐1‐B [38]	Grade 2 endometrial adenocarcinoma

### 3.2 GZD824 Inhibits Cell Migration and Invasion in Endometrial Cancer Cell Lines

**FIGURE 2:**
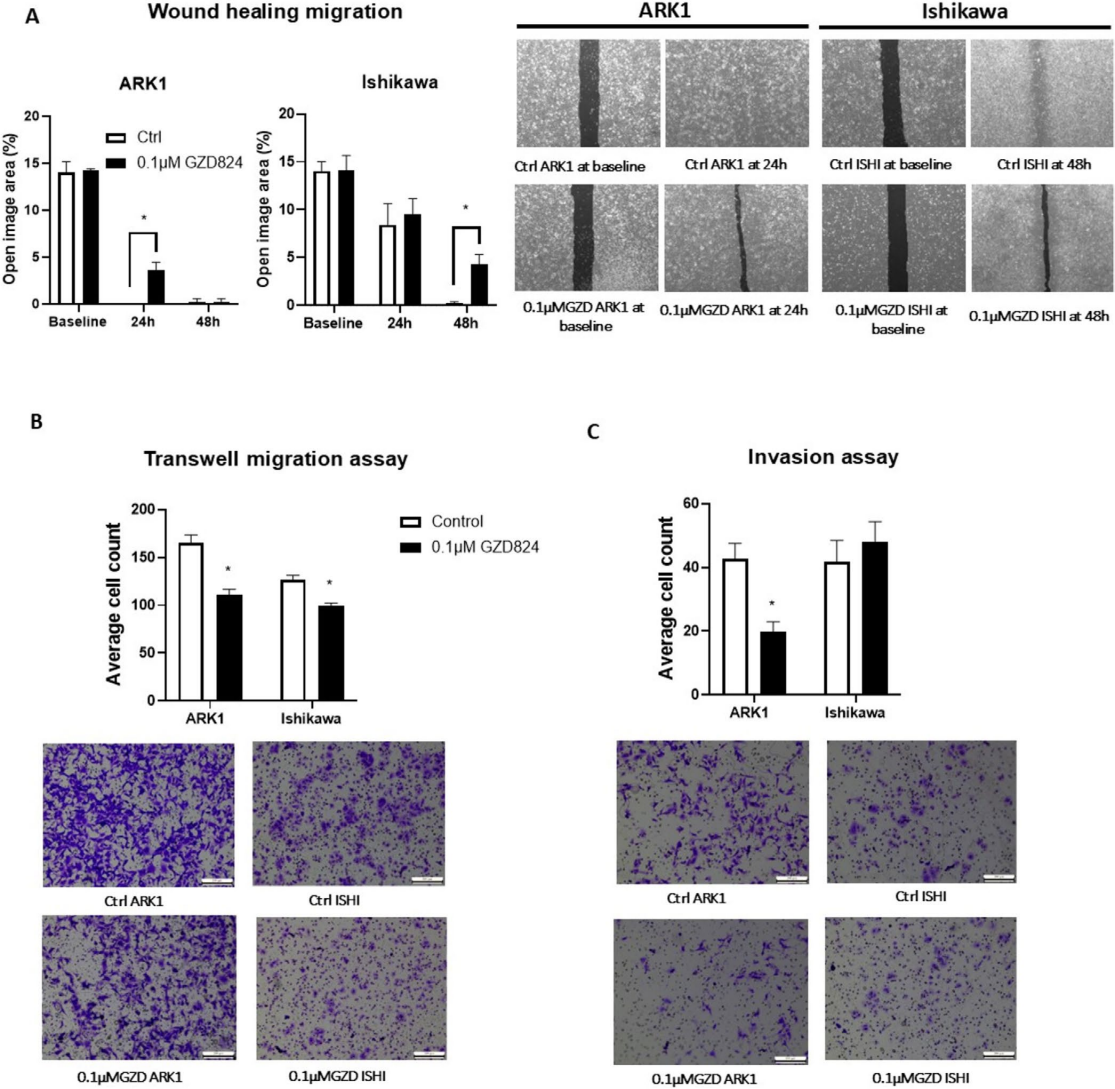
Effect of GZD824 on migration and invasion in Ishikawa and ARK1 cell lines. (A) Wound healing migration assays following treatment with 0.1 μM GZD824 in Ishikawa and ARK1 cells for 24 and 48 h. (B) Transwell migration assays following treatment with 0.1 μM GZD824 in Ishikawa and ARK1. (C) Transwell invasion assays following treatment with 0.1 μM GZD824 in Ishikawa and ARK1. For all panels *n* = 3, error bars represent standard deviation of the mean, *p < 0.05.

We next performed functional assays to evaluate the effect of GZD824 on cell migration and invasion ability of EC in vitro. Ishikawa and ARK1 were selected as models for endometrioid and serous EC subtypes, respectively. We treated cells with 0.1 μM GZD824 and observed significant inhibition of migration in both cell lines and invasion in ARK1 cells (Figure 2).

### 3.3 GZD824‐Mediated Regulation of Gene Expression and Downstream Pathways

**FIGURE 3:**
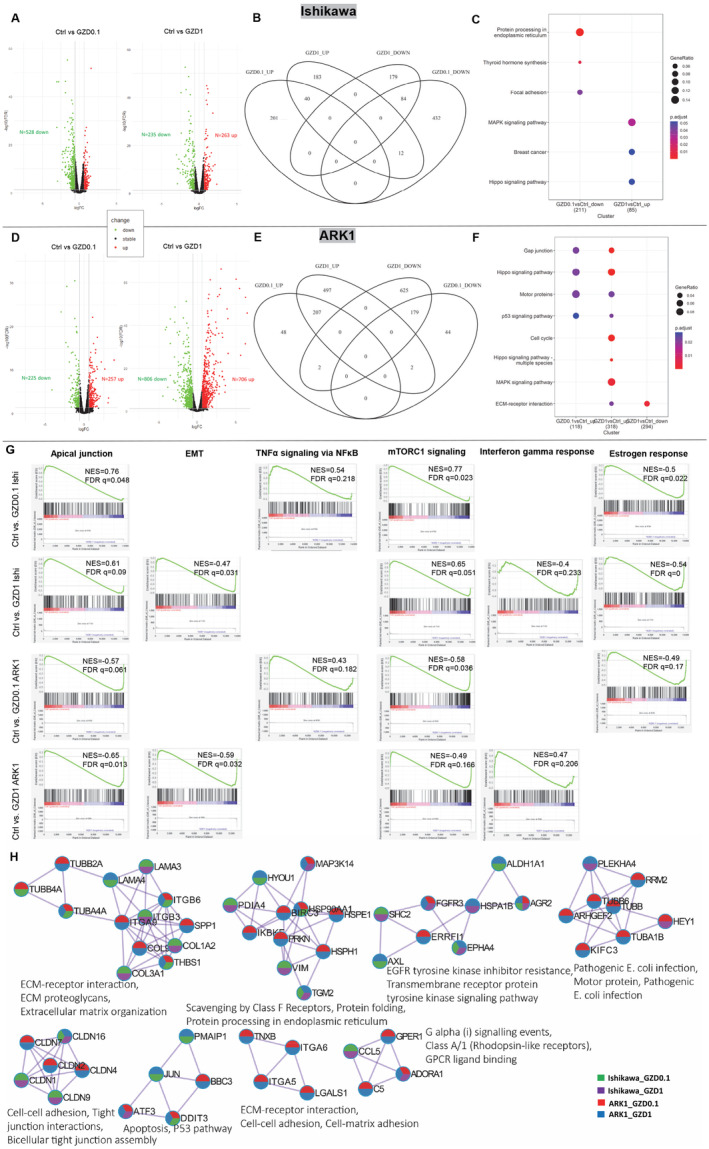
Gene expression and pathway analysis following GZD824 (0.1 and 1 μM) treatment of Ishikawa and ARK1 cells. Volcano plots present genes differentially expressed between GZD824 treatments and control in Ishikawa (A) and ARK1 (D) cells analysed by RNA‐seq. Venn diagram summarised the differential expressed genes overlapped between GZD824 treatment and vehicle control in Ishikawa (B) and ARK1 (E). Bubble plots of KEGG pathways analysed using differential expressed genes between treatments were shown for Ishikawa (C) and ARK1 (F). Significance is indicated by colour, and the number of genes is indicated by the size of the circle. (G) GSEA enrichment plots showed the hallmark pathways enriched in both Ishikawa and ARK1 following GZD824 treatments (normalised enrichment scores [NES], and the adjusted *p*‐values were labelled in each plot; details of enrichment results can be found in Table S4). (H) Enriched protein–protein interaction networks from Metascape. Nodes were represented by pie charts indicating their associations with each condition and colour coded gene lists. The top three most significant Gene Ontology enrichment terms for each MCODE cluster are annotated.

To investigate the impact of GZD824 on gene expression and downstream pathways, we performed RNA‐seq analysis of Ishikawa and ARK1 cells treated with GZD824 at 0.1 and 1 μM doses (IC70 doses for Ishikawa and ARK1 respectively). Following the GZD824 treatment, 241 genes were upregulated and 528 genes were downregulated at 0.1 μM dose while 263 were upregulated and 235 were downregulated at 1 μM in Ishikawa (Figure 3A, Table S1). In ARK1 cells, we observed 257 genes upregulated and 225 genes downregulated at 0.1 μM, while at 1 μM, 706 genes were upregulated and 806 genes were downregulated (Figure 3D, Table S1). Compared to the vehicle control‐treated Ishikawa cells, 40 genes were upregulated and 84 genes downregulated in both 0.1 and 1 μM doses, and 12 genes (*CILP*, *UPK3B*, *CYR61*, *SLC16A11*, *DUSP8*, *MUC15*, *ABCA1*, *CP*, *KHDRBS2*, *SLC26A7*, *GPC5* and *CREB5*) were downregulated in 0.1 but upregulated in 1 μM (Figure 3B). A similar pattern was observed in ARK1, with 207 genes showing upregulation and 179 genes displaying downregulation in both conditions, and 2 genes (*MYOM1* and *NFAM1*) upregulated in 0.1 but downregulated in 1 μM (Figure 3E). Among the differentially expressed genes (Table S1), we observed the downregulation of several downstream effectors of GCN2‐ATF4 signalling (e.g., *DDIT3*, *HSPA5*, *FGF19*, *TRIB3*, *CEBPG*, *ASNS*, *CEBPB*, *PMAIP1* and *PCK2*) [44]. In Ishikawa cells, this downregulation was particularly prominent at the highest GZD824 dose, while in ARK1 cells, effects appeared stronger at the lowest GZD824 dose.

We observed a number of KEGG pathways significantly enriched following GZD824 treatment in Ishikawa and ARK1 cells (Figure 3C,F). Specifically, MAPK and Hippo signalling pathways were enriched in both cell lines, while p53 signalling and cell cycle were enriched exclusively in ARK1. Within the top 100 Gene Ontology (GO) biological processes, P53 downstream pathway, IL‐18 signalling, Interleukin‐4 and Interleukin‐13 signalling, cell adhesion, PI3K‐Akt signalling pathway and regulation of programmed cell death were enriched across all the gene lists (Figure S1). Notably, the regulation of Rho GTPase, a critical modulator in response to the ROR1 initiated of the Wnt/planar cell polarity (PCP) signalling pathway [45], was exclusively impacted in ARK1 cells but not in Ishikawa cells.

Ingenuity Pathway Analysis revealed the enrichment of differentially expressed genes in pathways relevant to the actions of GZD824. For instance, identification of the Wnt/Ca^2+^ pathway in ARK1 (1 μM GZD824) and Ishikawa (0.1 μM GZD824) cells (Table S2) supported the targeting of ROR1 by GZD824. In concordance with observations in the GO biological processes, a number of Rho GTPase associated pathways were regulated in ARK1 (both 0.1 and 1 GZD824) but not in Ishikawa. Similarly, in both cell lines treated with 0.1 μM GZD824, the strong inactivation (*z*‐score ≥ |2|) of the unfolded protein response pathway, linked to GCN2 function [23], indicated the inhibition of GCN2 (Table S2). Moreover, we observed shared pathways across all treatments, including axonal guidance and gap junction pathways (Table S2). These pathways are known to be involved in cellular processes such as proliferation and migration [46, 47]. Notably, a number of pathways related to immune responses were also identified, including enrichment for Interleukin‐4 and Interleukin‐13 signalling (1 μM GZD824 in ARK1 and both doses in Ishikawa), PD‐1, PD‐L1 cancer immunotherapy pathway, IL‐6 and IL‐8 signalling (1 μM GZD824 in ARK1) and Immunogenic Cell Death Signalling Pathway (0.1 μM GZD824 in Ishikawa).

Ingenuity Pathway Analysis was also used to identify upstream regulators of differentially expressed genes. Consistent with the action of GZD824, GCN2 and ROR1 were found to be upstream regulators of differentially expressed genes in multiple GZD824 treatments (Table S3), while ATF4 emerged as a common upstream regulator across all treatments. A number of other upstream regulators were shared across all treatments and included those encoded by EC driver genes (e.g., *ARID1A*, *CTNNB1*, *FOXA2*, *TP53* and the PI3K and AKT families) [48]. Additional candidate EC GWAS susceptibility genes encoded upstream regulators, such as *WT1* [49] (Table S3), whose regulatory effects in Ishikawa cells (0.1 μM GZD824) were linked to the migration of tumour cell lines (Figure S2). Identification of upstream regulators also revealed some potential cell type specificity. For example, H2AX, whose phosphorylation is a marker of apoptosis, was found to be an upstream regulator specific to ARK1 cells, regardless of GZD824 doses (Table S3). PARP1 and caspase, whose cleavage are also markers of apoptosis, were also found to be an upstream regulator specific to ARK1 cells at 1 μM GZD824 dose (Table S3). Conversely, there was evidence for dose response effects on upstream regulators, with the oestrogen receptor showing diminished activation with increased GZD824 dose in both cell lines.

In addition, we performed the gene score enrichment analysis (GSEA) of hallmark gene sets following all GZD824 treatments (Figure 3G, Table S4). Apical junctions and mTORC1 pathways were enriched under all conditions. EMT and interferon gamma responses were significant following 1 μM GZD824 treatment, while TNFα‐ NF‐κB pathway was enriched at the 0.1 μM dose in both Ishikawa and ARK1. Oestrogen response was enriched in all conditions except for 1 μM GZD824‐treated ARK1. In ARK1 cells only, p53 pathway and IL2 STAT5 signalling were exclusively enriched, whereas the PI3K‐AKT pathway was enriched only in Ishikawa cells (Table S4).

To further explore the functional implications of the differentially expressed genes, PPI analysis was performed in Metascape [36] using all differentially expressed genes from Ishikawa and ARK1 cells. Eight MCODE clusters of the PPI were identified (Figure 3H). Processes including ECM‐receptor interactions, oestrogen signalling pathway, cell adhesion and DNA damage were enriched for differentially expressed genes from all sets of GZD824 treatment (Figure 3H, Table S5). The Wnt signalling pathway, EIF2AK1 response to heme deficiency and apoptosis were more significantly regulated in ARK1, whereas the response to hormone and response to growth factors were more significant in Ishikawa (Table S5).

### 3.4 GZD824‐Induced Protein Expression Changes Relate to Mesenchymal–Epithelial Transition, Cell Apoptosis and PI3K‐AKT Pathway in Endometrial Cancer Cell Lines

**FIGURE 4:**
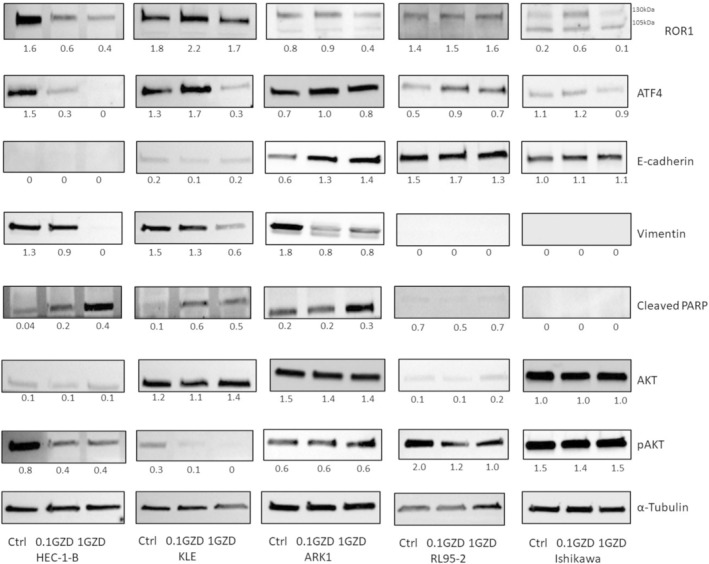
Western Blot results of key regulators in endometrial cancer cell lines HEC‐1‐B, KLE, ARK1, RL95‐2 and Ishikawa (ROR1 expression from high to low) following treatment with 0.1 and 1 μM GZD824. The numbers represent ratios of band intensity of protein versus its respective loading control (α‐Tubulin), calculated with ImageJ. For ROR1, only 130 kDa bands were quantified.

We examined the protein expression of key components in PI3K/AKT, GCN2/ATF4, epithelial–mesenchymal transition and apoptosis pathways in ROR1‐positive endometrial cancer cell lines HEC‐1‐B, KLE, ARK1, RL95‐2 and Ishikawa, following treatment with GZD824 for 48 h (Figure 4). Following treatment with 1 μM GZD824, there was a reduction in mature glycosylated ROR1 (130 kDa) expression in four out of five EC cell lines. Interestingly, an increase of the intensity of the mature glycosylated ROR1 protein was observed in four out of five cells treated with 0.1 μM GZD824. Three out of five cells showed a reduction in the key stress adaptation transcription factor ATF4 following 1 μM GZD824 treatment. Dual effects of the two doses on ATF4 were observed in KLE and Ishikawa cells, consistent with a previous study [22]. ROR1 high cells (HEC‐1‐B, KLE and ARK1) exhibited mesenchymal features, characterised by high levels of mesenchymal marker vimentin and low levels of epithelial marker E‐cadherin. These cells underwent mesenchymal to epithelial transition following both GZD824 doses, indicated by downregulation of vimentin. Increased levels of cleaved PARP were also observed in the three ROR1 high cell lines at both doses, suggesting activation of cell apoptosis. In addition, pAKT levels decreased in three out of five cell lines following GZD824 treatment, indicating downregulation of the PI3K‐AKT pathway.

## 4 Discussion

In this study, we investigated the therapeutic potential of GZD824 in EC by examining its effects on cellular phenotypes, gene and protein expression and downstream pathways. Firstly, to assess the cytotoxic effects of GZD824, we treated seven EC cell lines, as well as non‐cancerous cells derived from the endometrium or omentum. Consistent with a previous study in CLL [50], the cancer cell lines showed higher sensitivity to GZD824 compared to the non‐cancerous cells, highlighting its tumour specificity.

We focussed further analyses on Ishikawa and ARK1 cell lines, representative of endometrioid and serous endometrial tumours, respectively. We found that GZD824 inhibited cell migration in both cell lines, as well as invasion in ARK1 cells, indicating that it may be able to impair critical steps in EC metastasis. To gain insights into the molecular mechanisms underlying the effects of GZD824, we performed RNA‐seq analysis of GZD824‐treated Ishikawa and ARK1 cells. Results from analyses of differential expression, pathway enrichment and upstream regulators were consistent with the involvement of GCN2 in mediating effects of GZD824. Downregulation of GCN2‐ATF4 was observed in both Ishikawa and ARK1, with ARK1 showing higher sensitivity at a lower dose. This could be attributed to the higher levels of GCN2 (encoded by *EIF2AK4*) in ARK1 compared to Ishikawa, as indicated by our RNA‐seq data. The impact of GZD824 was more pronounced in ARK1 than in Ishikawa when administered at a higher dose. This led to alterations in both the downstream pathway initiated by ROR1 (Rho GTPase activity) and a reduction in the ROR1 protein level. In contrast, the Wnt/Ca^2+^ pathway was downregulated more significantly in Ishikawa cells. The Wnt signalling pathway, encompassing canonical (β‐catenin dependent) and non‐canonical (β‐catenin independent) arms, has been implicated in a range of malignancies including EC [40]. ROR1 primarily mediates the non‐canonical pathway by relaying signals from secreted Wnt glycoproteins to the intracellular Wnt/PCP and Wnt/Ca^2+^ pathways. Various initial events of ROR1 signalling have been reported in different contexts [11, 50, 51]. The variations in downstream pathways could be linked to the distinct ROR1 isoforms, which are correlated with their glycosylation status. Notably, ARK1 is predominantly expressed in the mature glycosylated form, while Ishikawa predominantly expresses the unglycosylated isoform. At the highest dose of GZD824, protein analyses provided additional support by demonstrating reduced ROR1 levels in ROR1 high EC cell lines (HEC‐1‐B, KLE and ARK1) and a loss of ATF4 expression in three out of five EC cell lines investigated. These findings collectively provide evidence for the role of ROR1 and GCN2 in the molecular responses to GZD824 treatment in EC, with potential context‐specific effects.

We identified shared upstream regulators across all GZD824 treatments, including those encoded by EC driver genes and candidate EC GWAS susceptibility genes [23]. These findings further highlight the involvement of such genes in key EC processes and their potential for therapeutic targeting. For example, we established a link between the upstream regulator WT1, encoded by a candidate EC GWAS susceptibility gene, and gene expression changes associated with tumour cell migration in GZD824‐treated Ishikawa cells. Moreover, pathway analysis revealed the enrichment of common pathways that may mediate the effects of GZD824 on proliferation and migration in both cell lines. Pathway and protein analyses also indicated that GZD824 may exert effects on PI3K/AKT signalling, where PI3K activation leads to AKT phosphorylation. This pathway has been implicated in EC cell invasion [52, 53, 54] and downstream signalling cascades of ROR1 [55]. Notably, the PI3K/AKT pathway has been identified as a promising target for treating EC due to its association with progestin [56] and cisplatin resistance [57] in EC. There have been a few drugs in EC clinical trials which target AKT that have shown effects [58].

Recently, immunotherapies, checkpoint inhibitors in particular, have provided new effective treatments for EC (reviewed in [59]). GZD824 has also been reported to activate immune responses within the tumour microenvironment and enhance the effects of a checkpoint inhibitor in renal cell carcinoma models [60]. Of relevance, we found pathways related to immunomodulation were enriched in both ARK1 and Ishikawa GZD824‐treated cells, suggesting another potential anti‐tumour activity mechanism for GZD824 in EC. Importantly, our findings suggest the potential combination therapy of GZD824 and immune checkpoint inhibitors in EC. These findings align with a recent report that GZD824 may enhance the infiltration of tumour‐infiltrating lymphocytes and potentiate responses to immune checkpoint inhibitors [60].

Targeting ROR1 has been reported to inhibit the EMT process [41], which plays critical roles in cancer progression and metastasis. Another major regulation following GZD824 treatment in ROR1 high EC cell lines (HEC‐1‐B, KLE and ARK1) involves the reversal of EMT, indicated by an elevated epithelial marker (E‐cadherin) alongside a downregulated mesenchymal marker (Vimentin). EMT events play critical roles in tumour progression as they confer metastatic characteristics upon cancer cells [61]. In contrast, in ROR1 low EC cell lines (RL95‐2 and Ishikawa), which exhibit epithelial phenotypes, the treatment had less impact. Therefore, GZD824 exhibits potential as a therapeutic option to inhibit metastasis in ROR1 high EC.

While our study provides valuable insights into the therapeutic potential of GZD824 in EC, several limitations should be acknowledged. Firstly, our analysis was conducted using in vitro experiments with cell lines, which may not fully recapitulate the complex tumour microenvironment present in vivo. Therefore, further studies using in vivo models or 3D models are necessary to validate our findings and assess the translational potential of GZD824. Secondly, the molecular mechanisms underlying the effects of GZD824 on EC cells are complex and multifaceted. Although we identified relevant pathways and upstream regulators that were shared across different GZD824 treatments, some findings would not survive correction for multiple testing. The elucidation of the molecular mechanisms that mediate the effects of GZD824 requires further in‐depth studies, including functional validation experiments. Thirdly, our analysis primarily focussed on two representative EC cell lines limiting the generalisability of our findings. Future studies should be performed with additional representative models to identify subtype‐specific effects, particularly for the rare subtypes of endometrial cancer that currently lack robust cell line models. Indeed, given the importance of four prognostic molecular subtypes established by The Cancer Genome Atlas and the Proactive Molecular Risk Classifier for EC in therapeutic decisions for EC (reviewed in [62]), it would be important to explore the effect of GZD824 in these subtypes.

In conclusion, our study demonstrates the therapeutic potential of GZD824 in EC. Furthermore, our findings suggest that GZD824 may have a role in suppressing metastasis and modulating immune responses within the tumour microenvironment. The expression changes observed in response to GZD824 support the targeting of ROR1 and GCN2, as well as the involvement of other relevant pathways and upstream regulators related to EC progression and susceptibility. However, further analysis and validation are required to reveal the precise molecular mechanisms that mediate the effects of GZD824 and translate findings into effective therapeutic approaches.

## Author Contributions

**Dongli Liu:** conceptualization (lead), formal analysis (lead), funding acquisition (lead), investigation (lead), methodology (lead), project administration (lead), visualization (lead), writing – original draft (lead). **Dylan Glubb:** formal analysis (supporting), investigation (supporting), methodology (supporting), resources (supporting), visualization (supporting), writing – review and editing (supporting). **Tracy O'Mara:** formal analysis (supporting), funding acquisition (supporting), supervision (supporting), writing – review and editing (supporting). **Caroline E. Ford:** conceptualization (supporting), project administration (lead), resources (lead), supervision (lead), writing – review and editing (supporting).

## Conflicts of Interest

The authors declare no conflicts of interest.

## Supporting information


**Table S1.** Differentially expressed genes from RNA‐seq analysis of Ishikawa and ARK1 cells treated with GZD824.

**Table S2.** Enriched pathways from the differentially expressed genes analysed by Ingenuity Pathway Analysis.

**Table S3.** Upstream regulators of differentially expressed genes identified via Ingenuity Pathway Analysis.

**Table S4.** Gene set enrichment analysis of the differentially expressed genes.

**Table S5.** Functional analysis of the differentially expressed genes using Metascape.

Data S1.

## Data Availability

The data that support the findings of this study are available in the Supporting Information of this article.
