# Supplementary material for: The Multi‐Kinase Inhibitor GZD824 (Olverembatinib) Shows Pre‐Clinical Efficacy in Endometrial Cancer
Source: Cancer Med. 2024 Dec 30;14(1):e70531. doi: 10.1002/cam4.70531 (PMC11683556; doi:10.1002/cam4.70531)
Supplement: Supplementary file 6 — Data S1. [file CAM4-14-e70531-s002.docx]

**Supplementary data**


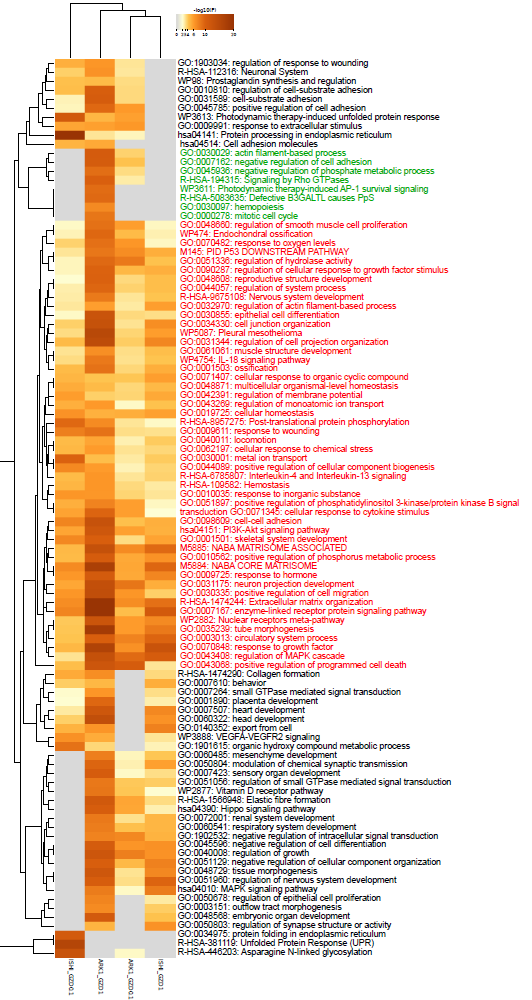


**Figure S1. Heatmap of top 100 Gene Ontology (GO) biological processes enriched across differentially expressed gene lists adjusted from Metascape.org.** Gray colour represented a lack of statistical significance. Pathways enriched in both conditions (0.1 and 1µM GZD824 treatments) of both Ishikawa and ARK1 were highlighted in red while those highlighted in green indicated the pathways exclusively modulated in ARK1.


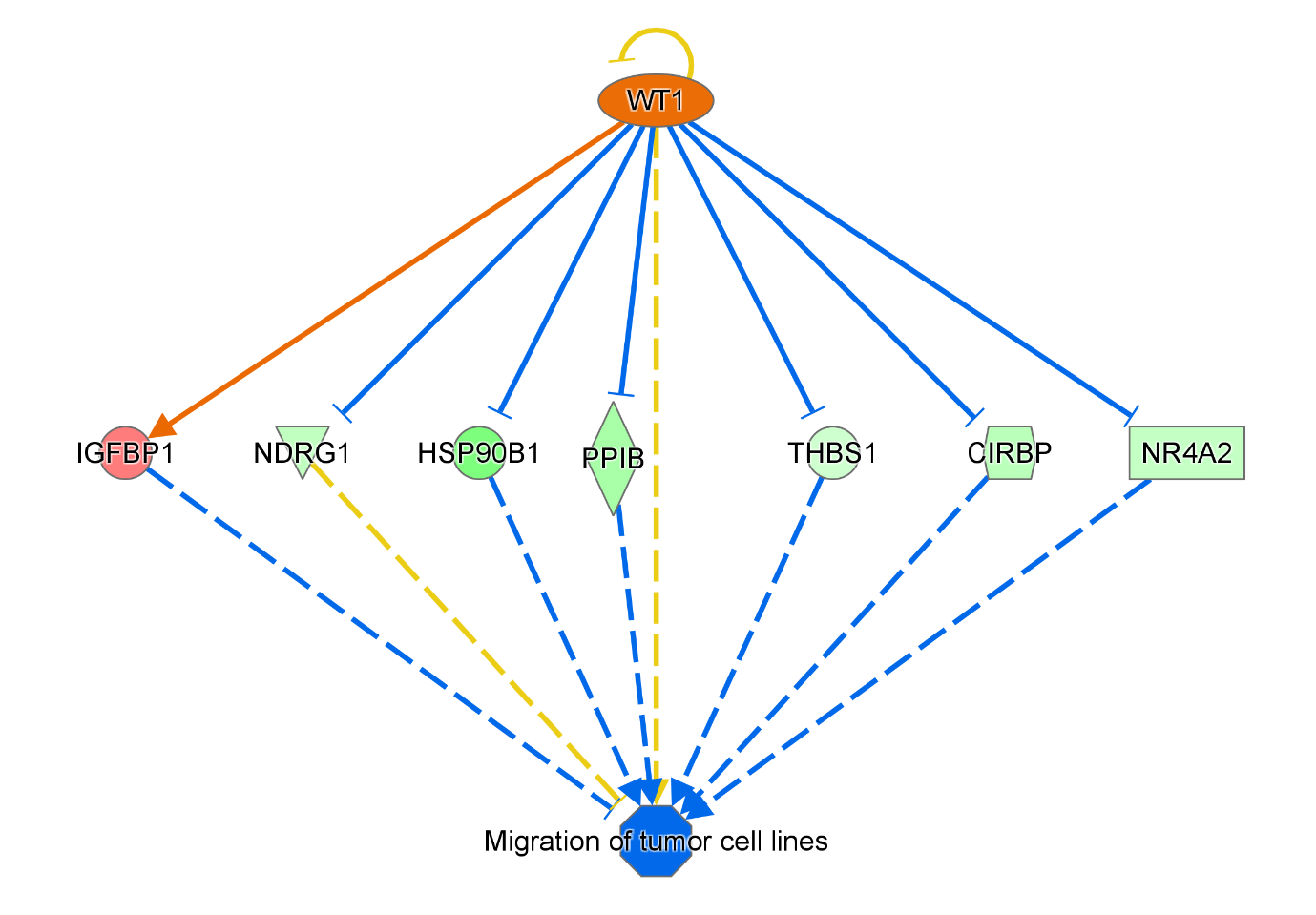


**Figure S2. Potential inhibition of tumour cell migration mediated through the upstream regulator WT1 in Ishikawa cells treated with 0.1 µM GZD824.** Predicted activation is represented by orange colours, while predicted inhibition is indicated by blue. The gold coloured lines denote findings that are inconsistent with the downstream state, and the dotted lines represent inferred relationships. The green colour of the WT1 gene targets indicates downregulation, while pink represents upregulation.
